# Target Classification Method of Tactile Perception Data with Deep Learning

**DOI:** 10.3390/e23111537

**Published:** 2021-11-18

**Authors:** Xingxing Zhang, Shaobo Li, Jing Yang, Qiang Bai, Yang Wang, Mingming Shen, Ruiqiang Pu, Qisong Song

**Affiliations:** 1 State Key Laboratory of Public Big Data, Guizhou University, Guiyang 550025, China; gs.xxzhang21@gzu.edu.cn (X.Z.); jyang23@gzu.edu.cn (J.Y.); yangwang@gzu.edu.cn (Y.W.); 2 School of Mechanical Engineering, Guizhou University, Guiyang 550025, China; cme.qbai18@gzu.edu.cn (Q.B.); 201407080@gznu.edu.cn (M.S.); gs.rqpu20@gzu.edu.cn (R.P.); gs.qssong18@gzu.edu.cn (Q.S.); 3 School of Mechanical & Electrical Engineering, Guizhou Normal University, Guiyang 550025, China

**Keywords:** tactile sensor, tactile perception data, ResNet, target classification

## Abstract

In order to improve the accuracy of manipulator operation, it is necessary to install a tactile sensor on the manipulator to obtain tactile information and accurately classify a target. However, with the increase in the uncertainty and complexity of tactile sensing data characteristics, and the continuous development of tactile sensors, typical machine-learning algorithms often cannot solve the problem of target classification of pure tactile data. Here, we propose a new model by combining a convolutional neural network and a residual network, named ResNet10-v1. We optimized the convolutional kernel, hyperparameters, and loss function of the model, and further improved the accuracy of target classification through the K-means clustering method. We verified the feasibility and effectiveness of the proposed method through a large number of experiments. We expect to further improve the generalization ability of this method and provide an important reference for the research in the field of tactile perception classification.

## 1. Introduction

Research on object classification based on tactile perception data is much less than that based on visual image data. However, tactile perception is better than vision in processing the material characteristics and detailed shapes of a target, especially in poor-light environments [1,2,3,4]. Tactile sensor technology and the continuous development of deep-learning processes promote interdisciplinary research robot target recognition [5,6]. The target classification of tactile data is widely used in the operation of humanoid robots, which has important practical significance for the development of robotics.

In recent years, tactile sensor technology has rapidly developed, and there have been many advances in performance and applications [7,8,9]. The tactile sensor technology can detect the force of a target in real time, and apply detected tactile pressure data to the target recognition problem [10]. Alin Drimusa, Gert Kootstrab et al. [7] demonstrated the application of a new type of tactile array sensor based on flexible piezoresistive rubber in an active target classification system. The authors based it on the k-nearest neighbor classifier, which uses dynamic time warp to calculate the distance between different time series that can successfully identify the target. Zhanat Kappassov, Daulet Baimukashev et al. [8] designed a series elastic tactile array of 16 sensor elements arranged in 4 × 4 to realize the tactile exploration of the position control robot manipulator. The authors proved the sensor’s tactile exploration capabilities through classification experiments on deformable rigid targets. Elliott Donlon et al. [9] proposed a high-resolution tactile finger for robotic grasping. The finger sensor outputs an image of the tactile imprint to encode the shape and texture of the object at the contact. This image information can be applied to model-based object classification and robot grasping.

In addition, relevant domestic and foreign researchers in the field of artificial intelligence proposed many classification methods based on deep learning in their recent work to obtain better target classification accuracy [11,12,13,14,15,16]. Marianna Madry et al. [13] proposed a spatiotemporal hierarchical matching pursuit (ST-HMP) unsupervised feature learning method. The ST-HMP method can extract rich spatiotemporal structures from raw tactile data without predefining distinguishing data features. The authors applied it to grasping stability evaluation and object instance classification. The authors verified using multiple synthetic and real datasets collected by Schunk-Dexterous, Schunk-Parallel, and iCub-hands. Subramanian Sundaram et al. [14] built a deep convolutional neural network model to process and analyze tactile data, but the training effect of the proposed method was not very satisfactory, and the highest classification accuracy was only 77.67%. Chunfang Liu et al. [15] proposed a spatiotemporal tactile representation framework for target recognition with the advantages of spatiotemporal modeling, nonlinear coding, and efficient codebook format, and a new efficient codebook formula clustering method (LDS- FCM). Then, the final feature description of the tactile data was derived using the VLAD method, and verified by 5 public databases (BDH5, SPR7, SPR10, SD5 and SD10). Satoshi Funabashi et al. [16] studied the problem of tactile target recognition with relatively densely distributed force vector measurement, and analyzed the tactile information that is conducive to target recognition and classification. The UsKin tactile sensor was embedded in Alelgo’s hand, and a total of 240 three-axis force vector measurements are provided in all fingers to obtain time-series training and test data. Simple feedforward, recursive, and convolutional neural networks are used to identify targets. The recognition rate of 20 targets can be as high as 95%. The evaluation shows that high-dimensional information provided by the sensor is indeed beneficial for target classification [16].

The above methods are all studies on the object classification problem of pure tactile perception data that have obtained good classification accuracy. However, due to the complexity of the tactile sensory data characteristics of targets of different sizes, shapes, and hardness levels, most of the current studies are limited to the classification problem of less than 20 types of targets and a small amount of sensor data. When dealing with the classification problem of complex tactile perception data based on more targets, the training effect of these algorithm models is often unstable, easily falls into overfitting, and cannot achieve the expected classification accuracy.

In response to the above problems, we propose a target classification model based on pure tactile perception data. Our model utilizes the advantages of convolutional neural networks and deep residual networks in feature learning. First, we converted one-dimensional information collected by the tactile sensor into a 32 × 32 tactile map as the input of the model. Second, we continuously optimized the model to achieve the expected classification effect. Lastly, we verified the effectiveness and feasibility of our model through a large number of experiments. The main contributions of this paper are as follows.

(1) The convolutional neural-network algorithm model is applied to more than 20 types of object classification problems based on the tactile perception data of multi-tactile sensors, and the effective application in more complex tactile perception capture data is realized.

(2) By increasing the number of sensors, the complexity of capturing data is increased, thereby increasing the tactile perception grasping characteristics, so that the manipulator can better learn human grasping characteristics.

(3) We proposed and optimized an improved residual network model (ResNet10-v1) to improve the accuracy of multi-objective classification for complex tactile perception data. The accuracy rate of the highest category reached 80.10%, and the highest accuracy of the three categories was 92.72%.

This rest of the paper is organized as follows: Section 2 describes our proposed classification method for tactile perception targets. Section 3 presents the experimental results, analysis, and discussion. Section 4 summarizes the research work and discusses future research directions.

## 2. Proposed ResNet10-v1 Architecture

**Figure 1:**
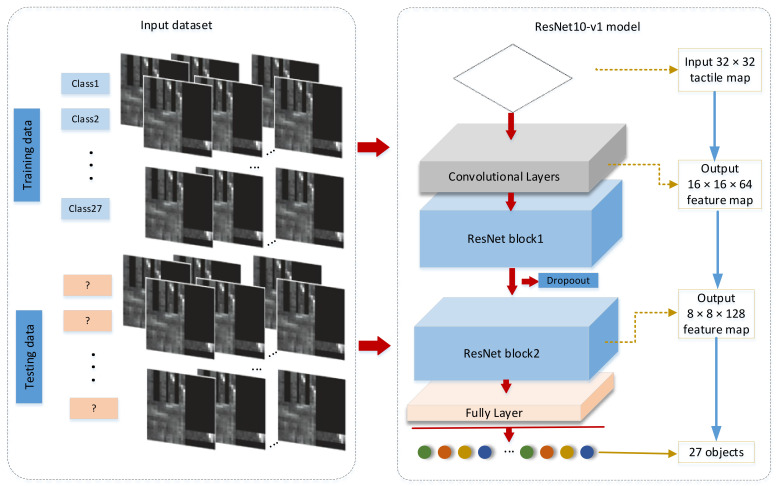
Proposed ResNet10-v1 structure.

Aiming at the target classification problem of tactile data with complex features, our proposed ResNet10-v1 architecture is shown in Figure 1. In this model, we converted one-dimensional information collected by the tactile sensor into a 32 × 32 tactile map as the input of the ResNet10-v1 model.

The model consists of a total of 10 layers of networks (containing only convolutional and fully connected layers, and not normalization and pooling layers). The input data pass through convolutional, batch normalization, ReLU, and maximal pooling layers, two ResNet blocks, and lastly a fully connected layer to output the target type. 

The convolutional layers are convolutional, batch-normalization, ReLU, and maximal pooling layers. The input data are convolved with the filter kernel in the convolutional layer, and the convolutional process is described in Equation (1).

(1) $$y_{i}^{l+1}(j)=K_{i}^{l}*x^{l}(j)+b_{i}^{l}$$

where $K_{i}^{l}$ represents the weight, and $b_{i}^{l}$ represents the offset of the *i*-*th* layer filter in the *l*-*th* layer. We use $x^{l}(j)$ to represent the *j*-*th* partial area in the l layer, and * is used to calculate the dot product of the kernel and the partial area. The input of the convolutional layer is a tactile map reflecting the pressure data of different targets.

The main function of the batch-normalization layer is to standardize data [17]. It has the advantages of improving the generalization ability of the trained network and avoiding the influence of singular data on the model. Batch normalization can normalize the tactile pressure value between 500 and 1024, and between 0 and 1.

The ReLU function is simple to calculate and can speed up model training. The most important thing is that the classification problem can be mapped into a nonlinear problem to improve the effectiveness of the model.

The pooling layer is an important part of the CNN classifier, and its working method is to gradually reduce the space size of the representation [18,19]. Since input tactile map data need to quickly reduce dimensionality, we designed the largest pooling layer to reduce the size of the tactile map.

The pooling layer can reduce the scale of the convolutional neural network model, increase the speed of model calculation, and improve the robustness of feature extraction. In maximal pooling, the largest element in each pooling area is selected and defined by Formula (2).

(2) $$p_{k,(i,j)}=\underset{(p,q)\in Q_{\left(i,j\right)}}{\max}(R_{k,(p,q)})$$

where $p_{k,(i,j)}$ is the output of the pooling operator related to the *k*-*th* feature map, $R_{k,(p,q)}$ is the element at position (*p, q*) in the pooling area, and $Q_{\left(i,j\right)}$ represents the pooling area around position (*i, j*).

The residual network model is composed of many superimposed residual (ResNet) blocks. Compared with a conventional neural network, the residual network has one more direct channel that can skip the middle layer and directly reach the state before output [20,21,22]. From the perspective of feature extraction, the network combines shallow and deep features to predict and judge, which increases the complexity of the features and effectively avoids the problem of gradient disappearance.

In order to prevent the problem of the training effect being good but the test effect being poor, that is, the problem of overfitting, we added a dropout layer between the two ResNet blocks. During the training process, a certain percentage of neurons (usually 0.3 or 0.5) are randomly discarded.

Our model combines the advantages of a typical convolutional neural network and a deep residual network structure, and achieves the expected effect of target classification.

### 2.1. Improvement of Convolutional Kernel

**Figure 2:**
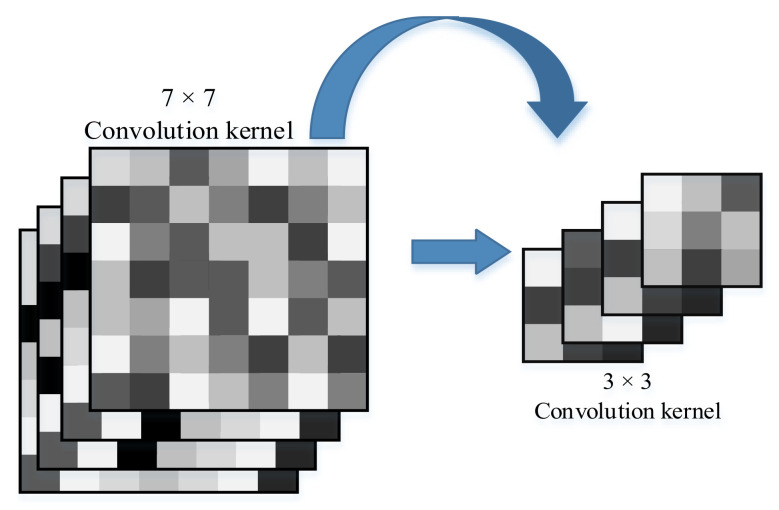
Different kinds of convolutional kernels.

On the basis of the Resnet18 structure, we modified the convolutional kernel filter of the convolutional layer before the data are input to the residual block. We changed the 7 × 7 convolutional kernel into a 3 × 3 convolutional kernel, as shown in Figure 2. 

In addition, we changed the stride of the first convolutional layer of the original Resnet18 from 2 to 1 because the width of each finger of the tactile glove was 3 pixels (that is, the pressure data of 3 tactile sensors), which maps the smallest feature of the sensor data. This is in order for the convolutional kernel to adapt to the smallest features in the sensor data.

### 2.2. Adaptive Optimization of Learning Rate

The learning rate is the amount of the weight update in the network during the training phase [23,24], which indicates that it is an important hyperparameter for the successful application of the ResNet10-v1 model. The constant learning rate cannot meet the iterative needs of the model training in the early, mid, and late stages. Therefore, we adaptively improved the learning rate as shown in Equations (3)–(5) to meet the learning rate in different periods. Requirements:

(3) $$g=0.1^{\left(1.0/p\right)}$$

(4) $$New\_lr=Base\_lr\times g^{epoch}$$

(5) $$New\_lr=Base\_lr\times {\left(0.1^{\left(1.0/p\right)}\right)}^{epoch}$$

where *P* is set to a constant of 1000, *Base_lr* represents the initial learning rate, *New_lr* represents the updated learning rate, and epoch represents the number of times that the model is fully trained (including one forward pass and one back pass) using all samples in the training set. Formulas (3) and (4) are combined to obtain final adaptive Formula (5). The calculation method of Formula (5) continuously iterates the new learning rate, so that *New_lr* decreases with the increase in epoch to satisfy the convergence of the ResNet10-v1 model to the local minimum.

### 2.3. Cross-Entropy Improvement 

The loss function used by the ResNet10-v1 model is cross-entropy, and the implementation formula is as follows.

(6) $$L=\frac{1}{N}\sum_{i}L_{i}=\frac{1}{N}\sum_{i}-\sum_{c=1}^{M}y_{ij}\log(p_{ij})$$

where *M* is the number of target categories, and $y_{ij}$ represents the actual class. When the *i*-*th* sample belongs to category *j*, the value of $y_{ij}$ is 1; otherwise, it is 0. $p_{ij}$ represents the predicted probability of the *i*-*th* sample belonging to class *j*, and *i* represents the *i*-*th* sample.

### 2.4. K-Means Cluster Analysis Method

Clustering algorithm refers to clustering a group of targets that are more similar to each other in a certain characteristic. Targets belonging to the same class are divided into a group, which is called a cluster [25,26]. Cluster analysis is a method that can be used for feature-level fusion [27]. In the cluster analysis method, the K-means algorithm is widely used in large-scale data target recognition.

**Figure 3:**
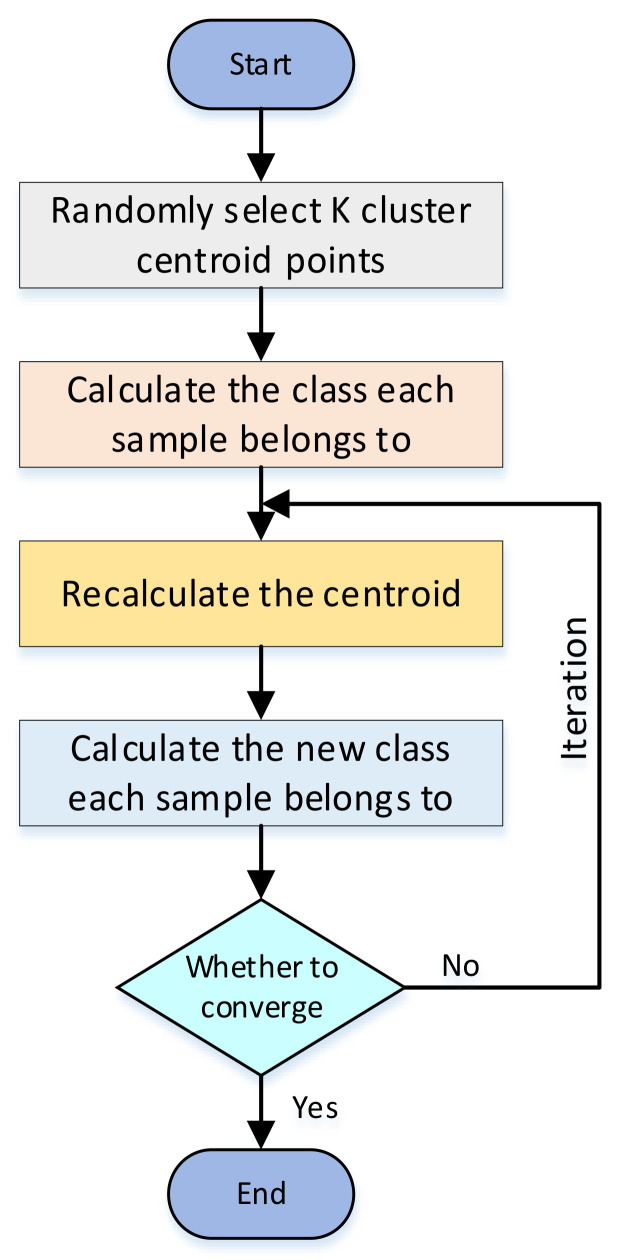
Principle of K-means algorithm.

The K-means algorithm clusters samples into k clusters. Because of its advantages of speed and simplicity [28], we used classical K-means clustering in the clustering method to cluster tactile datasets collected by different grasping methods of the target, and classify the target after clustering. This increases the effective features of tactile sensing capture data and helps in improving the classification accuracy of multiple types of targets. The basic principle of the K-means clustering algorithm is shown in Figure 3.

First, K cluster centroid points are randomly selected, and the class to which each sample belongs is calculated according to the principle of minimal distance, that is, the shortest distance between sample and K centroid points. After many iterations, whether the position of the centroid point does not change or only changes a little is judged, that is, whether the distance between the centroid points of the previous and next generations converges. If it does not converge, the centroid iteration continues looping and reclustering. If it converges, the iteration ends, and the clustering of K-type targets is achieved. 

To determine whether the best clustering result is achieved, a distortion function J needs to be introduced, as shown in Formula (7). The distortion function represents the sum of the squares of the distance between each sample and its corresponding centroid point. When the distortion function reaches the minimal value, the clustering effect reaches the theoretically best result.

(7) $$J(c^{\left(i\right)},\mu_{c^{i}})=\frac{1}{n}{\sum_{i=1}^{n}\left\|x^{\left(i\right)}-\mu_{c^{\left(i\right)}}\right\|}^{2}$$

where $\mu_{c^{\left(i\right)}}$ represents the centroid point of the cluster closest to sample point $x^{\left(i\right)}$, and $c^{\left(i\right)}$ represents the distance between $\mu_{c^{\left(i\right)}}$ and $x^{\left(i\right)}$. The K-means algorithm is to find the smallest $c^{\left(i\right)}$ and $\mu_{c^{\left(i\right)}}$, so that distortion function J reaches the minimum.

When grasping targets in different ways, the greater the difference in pressure information distribution and the more obvious the grabbing features are, the easier it is for the classification model to learn to make better predictions. Therefore, we adopted a two-step cascade method, that is, the K-means clustering algorithm and the ResNet-v1 model were used in tandem. First, we input the pressure data of 26 types of targets with different capture methods into the K-means algorithm for clustering. Then, we randomly divided the data output by the clustering algorithm using it as the input data of the ResNet-v1 model, and further identified the target.

### 2.5. Basic Unit Settings of Network Layer and Output Data Dimensions

**Figure 4:**
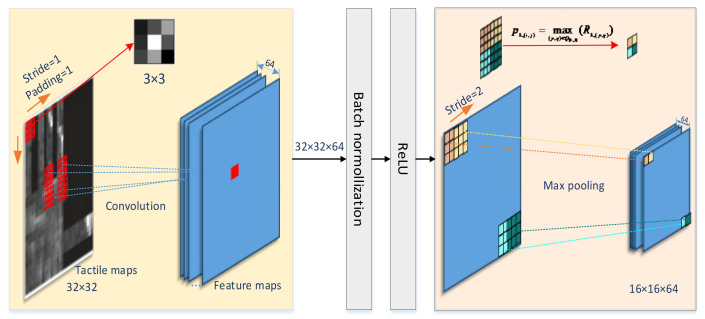
Convolutional layer principle.

The input layer size accepted by the ResNet10-v1 model is 32 × 32. As shown in Figure 4, the size of the convolutional kernel of the convolutional layer was 3 × 3, padding was 1, and the stride was 1. Since all 0 padding was used, after the convolutional layer, the output size was still 32 × 32.

The input of the max pooling layer is the output of the previous layer, which is a 32 × 32 × 64 node matrix. The filter size that we designed was 3 × 3, stride = 2, so the node matrix with a size of 32 × 32 × 64 can be reduced to 32/2 × 32/2 × 64 = 16 × 16 × 64 data after the pooling layer. Since the model separately performs the max pooling operation on each channel, the number of channels after pooling is the same as the number of input channels. Using the pooling layer both speeds up the calculation and prevents overfitting.

After two ResNet blocks, the data size changed from 16 × 16 × 64 input to 8 × 8 × 128 output. The depth increased, and dimensionality decreased. Then, after the average pooling layer, data were averaged and flattened into a one-dimensional vector with a length of 128.

Each node of the fully connected layer was connected to all nodes of the previous layer, and was used to integrate extracted features from the front. There were 128 fully connected input nodes and 27 output nodes. Since the classification target was 27 categories, the output node was 27. Total parameters were 128 × 27 + 27 = 3483.

## 3. Experimental Results and Analysis

In our experiments, all calculations were performed using a computer with an 8 GB GPU (NVIDIA GeForce GTX 1660) and a Windows 10 operating system. Python was used with the Keras and Pytorch frameworks to implement the target classification problem on the basis of convolutional residual networks.

### 3.1. Experimental Setup

**Figure 5:**
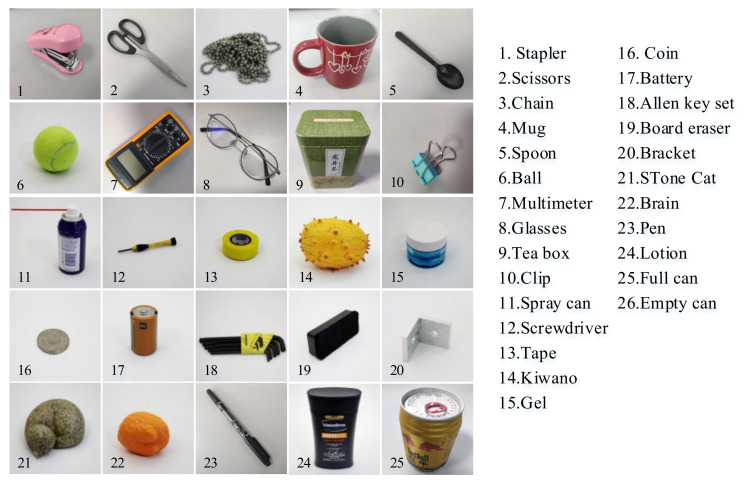
Dataset objects [14]; 26 targets used in our experiments.

**Figure 6:**
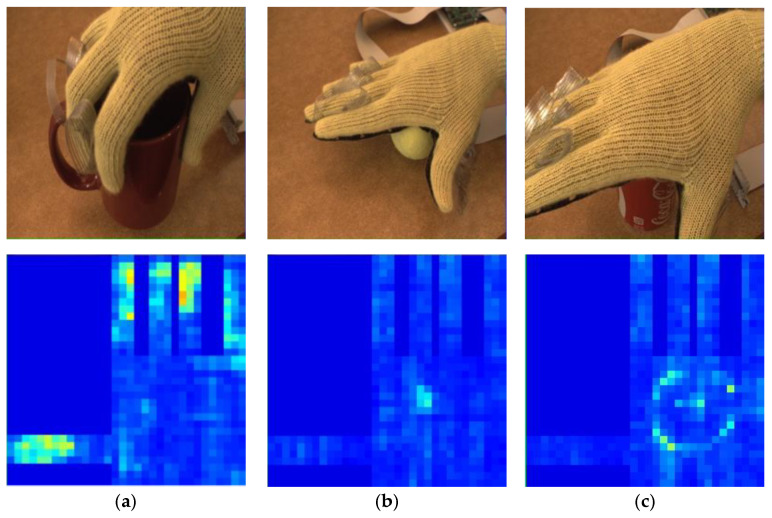
Tactile maps obtained when tactile glove grabs different targets. (**a**) Cup; (**b**) tennis ball; (**c**) cola can.

In order to verify the performance of our convolutional neural network model in the object classification problem of tactile perception data, we chose the public dataset of the Massachusetts Institute of Technology Computer Science and Artificial Intelligence Laboratory as the original [14]. This dataset was obtained by grasping experiments on 26 types of targets (Figure 5) with a tactile glove with 548 tactile sensors on the entire hand. Tactile perception data were recorded by 548 tactile sensors during the grasping process. Each group of data was processed into a 32 × 32 tactile map that mapped all sensor data. These tactile maps (Figure 6) were input into the ResNet10-v1 model proposed in this paper for training.

The dataset contained 27 categories that are the tactile perception data of 26 targets of different shapes, sizes, and hardness levels, and a category of data captured with bare hands. The number of samples in each category was 2000 32 × 32 tactile maps, and the training, validation, and test samples were divided according to the ratio of 7:1:2. The training samples included 1400 32 × 32 tactile-map data of various targets (1400 × 27 = 37,800 tactile maps in total). The validation samples included 200 32 × 32 tactile-map data of various targets (200 × 27 = 5400 tactile maps in total), and the test samples included 400 32 × 32 tactile-map data of various targets (400 × 27 = 10,800 tactile maps in total). If there were samples in the test set that were not in the training set, and the model was unable to classify the target, they were judged as another class.

### 3.2. Comprehensive Evaluation Index

This article uses Val-top1, Val-top3, Val-cluster-top1 and Val-cluster-top3, test-top1, test-top3, test-cluster-top1 and test-cluster-top3 as the evaluation indicators for model classification.

Val-top1 represents the accuracy of the target class with the highest classification effect after verifying the model on the validation dataset.

Val-top3 represent the probability that all targets belong to the top three in classification accuracy on the validation dataset.

Val-cluster-top1 represents the verifying accuracy of the target class with the best classification effect after K-means clustering.

Val-cluster-top3 represents the probability that all targets belong to the top three in classification verifying accuracy after K-means clustering. 

Test-top1 represents the accuracy of the target class with the highest classification effect after testing the model with the testing dataset.

Test-top3 represents the probability that all targets belong to the top three in classification accuracy on the testing dataset.

Test-cluster-top1 represents the testing accuracy of the target class with the best classification effect after K-means clustering.

Test-cluster-top3 represents the probability that all targets belong to the top three in classification testing accuracy after K-means clustering.

In order to avoid contingency and reduce errors, we carried out 10 repeated experiments for each set of parameters, and the final classification result was obtained by averaging the results of the 10 experiments. The average calculation formula is shown as Formula (8).

(8) $$\bar{x}=\frac{1}{n}\sum_{i=1}^{n}x_{i}$$

where $x_{i}$ refers to the accuracy rate obtained in the *i*-*th* experiment (*i* = 1, 2, …, *n*, *n* = 10), and $\bar{x}$ refers to the average accuracy rate of 10 experiments.

### 3.3. Hyperparameter Optimization Results and Analysis

The choice of hyperparameters requires continuous experiments to obtain better results. In order to find the relative optimal values of various hyperparameters, this section optimizes the main hyperparameters of the model (such as learning rate, epoch, Batch_size, dropout), and analyzes and summarizes the optimization results.

#### 3.3.1. Base Learning Rate

In order to find a better initial learning rate, we conducted six sets of experiments using the ResNet10-v1 model. They are the obtained classification accuracy rates when the initial learning rate (Base LR) was 10^−1^ 10^−2^, 10^−3^, 10^−4^, 10^−5^, or 10^−6^.

The basic parameter settings of the six groups of experiments were as follows: Epoch = 1, Batch_size = 32, input nframes = 3. Each experiment was carried out 10 times.

**Figure 7:**
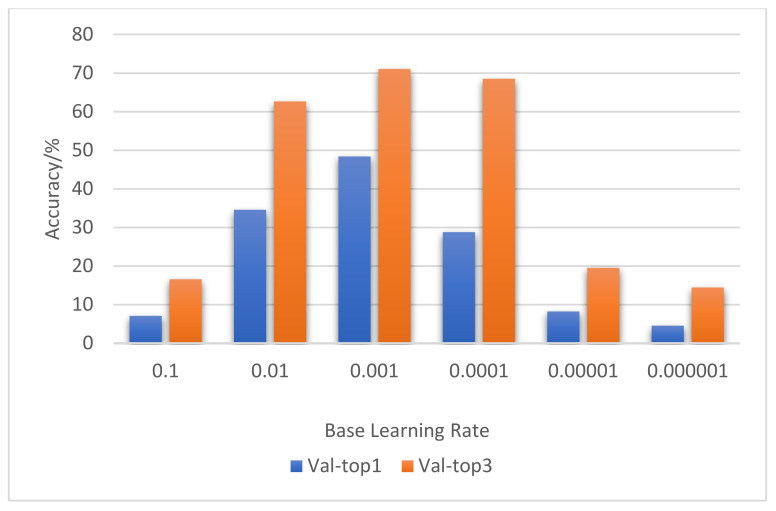
Result comparison of base learning rate optimization.

Experimental results in Figure 7 show that, when the initial learning rate was equal to 10^−1^, 10^−2^, or 10^−3,^ the accuracy rate gradually increased. However, when the initial learning rate was equal to 10^−4^, 10^−5^, or 10^−6^, the accuracy rate gradually decreased. When the initial learning rate was optimized to 10^−3^, the prediction accuracy rate was the highest on the validation data.

#### 3.3.2. Epoch Optimization 

Epoch refers to the amount of the entire dataset that is passed through the network only once in the deep-learning classification model [29]. As an important hyperparameter, it is necessary to determine the optimal epoch value for a given dataset. Therefore, we continuously optimized the value of epoch to obtain its best value.

**Figure 8:**
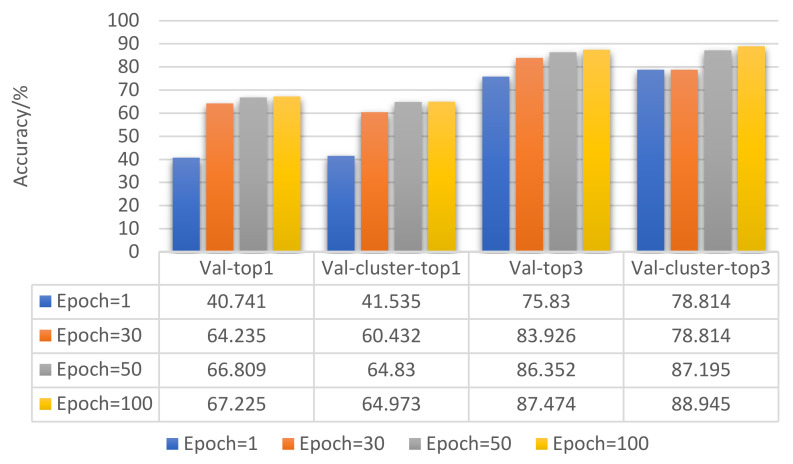
Result comparison of epoch optimization.

The experiment was divided into four groups: epoch = 1, epoch = 30, epoch = 50, and epoch = 100. Ten experiments were performed for each group of experiments, and the average value was calculated according to Formula (8). Figure 8 shows the comparison of the results after 10 experiments were averaged.

Figure 8 shows that, as the epoch increased, the accuracy of the model’s validation on the validation set gradually increased. However, the overall trend of its growth gradually slowed down. Epoch = 100 was the best value for model training.

The basic parameter settings of the four groups of experiments were as follows: base LR = 10^−3^, batch_size = 32, input nframes = 7.

#### 3.3.3. Batch_size Optimization

Batch_size represents the number of training samples that pass through the network at one time. In order to find the best balance between memory efficiency and capacity, it is necessary to optimize Batch_size and choose a relatively optimal Batch_size. For a normal dataset, if Batch_Size is too small, it is very difficult for the training data to converge, resulting in underfitting.

**Figure 9:**
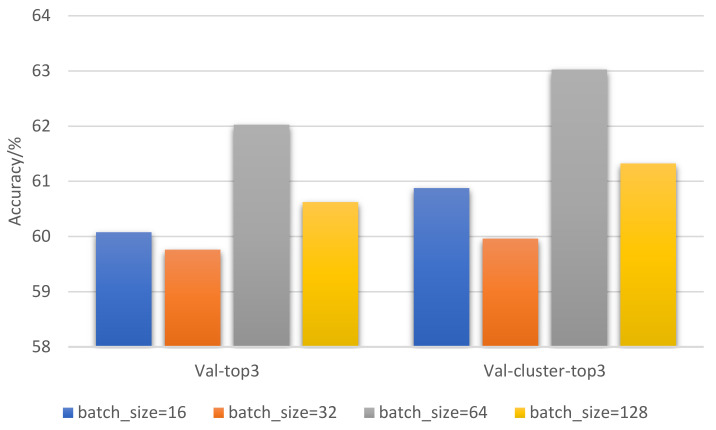
Result comparison of Batch_size optimization.

In order to improve the accuracy of model prediction, we set batch_size to 16, 32, 64, 128, and 256 to conduct five sets of experiments. Each set of experiments is performed 10 times and the results are averaged. The experimental settings are as follows: epoch = 30, nframes = 1, base LR = 10^−3^. The comparison of Batch_size optimization results is shown in Figure 9: Batch_size = 64 was the set of experiments with the best target classification effect.

#### 3.3.4. Dropout Optimization 

When training a convolutional neural network model, the problem of overfitting often occurs, that is, the prediction accuracy rate on the training sample is high, and the prediction accuracy rate on the test sample is low [30]. Adding a Dropout layer to the model can relieve the network from overfitting, and the dropout loss rate needs to be tried and selected according to specific networks and specific application areas.

**Figure 10:**
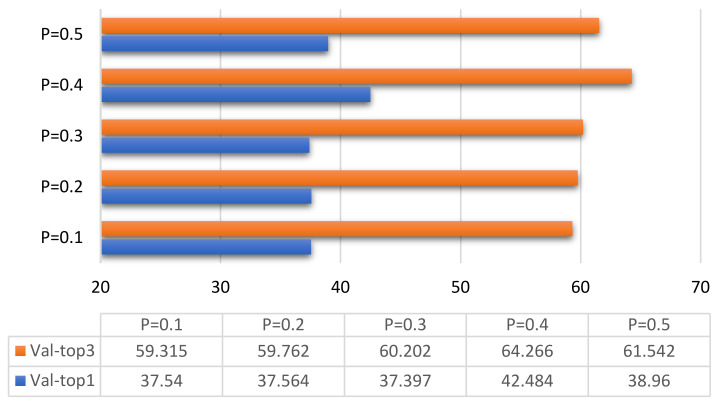
Result comparison dropout optimization.

In order to study the influence of the Dropout layer on the classification of the ResNet10-v1 model and find a network model suitable for the classification of tactile perception data, we only consider one Dropout layer with different loss probability values. A total of six loss probabilities P are considered: 0.1, 0.2, 0.3, 0.4, 0.5, and other hyper-parameters remain unchanged, and Dropout is optimized to achieve the best effect. The optimized comparison result is shown in Figure 10.

Figure 10 clearly shows that, when dropout loss ratio *P* = 0.4, Val-top1 was 42.484%, and Val-top3 reached 64.255%. The training and validation effects of the ResNet10-v1 model for tactile perception data were much better than those when *P* = 0.1, *P* = 0.2, *P* = 0.3, and *P* = 0.5.

### 3.4. Optimization of Number N of Input Dataset Categories

**Figure 11:**
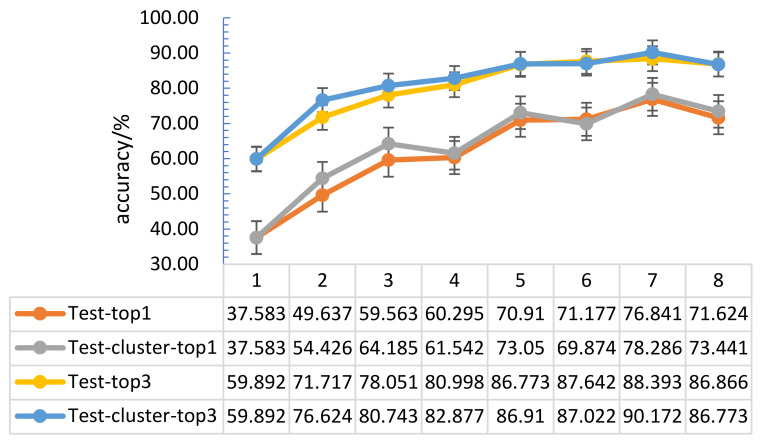
Optimization result comparison chart of different capture method datasets.

The tactile data obtained through only one kind of grasping method show that the tactile perception characteristics were not prominent, and the training effect was poor. In order to increase the number of effective features of the tactile perception data and achieve a better target classification effect, it is necessary to use a variety of methods to capture the target. This section studies the tactile perception data of categories 1 to 8 with similar grasping methods. Here, the number of input dataset categories is denoted by N, and the 32 × 32 tactile map formed by the collected tactile data was input into the convolutional neural network model. The 26 obtained target classification results are shown in Figure 11.

Figure 8 shows that, when using N different tactile datasets with different grasping methods as input, compared with randomly selecting one of the input, the target recognition accuracy was significantly improved; when N = 1, 2, 3, 4, 5, 6, 7, the recognition accuracy of the target showed an overall upward trend. When N = 8, there were some redundant data, which led to the problem of target recognition confusion, so the target recognition accuracy rate dropped. Experiments show that the accuracy of target recognition increased as the number of input categories increased, and reaches its best performance with about 7 random input frames.

In order to better compare the optimization effect of our convolutional residual network model, we combined relatively good hyperparameters (epoch = 200, base LR = 10^−3^, batch_size = 64, dropout = 0.4 and N = 7), and conducted many experiments to compare and analyze the accuracy of model classification before and after optimization.

The comparison results of the proposed model before and after optimization are shown in Table 1. The experimental hyperparameter settings after model optimization are as follows: base LR = 10^−3^, Batch_size = 64, epoch = 200.

**Figure 12:**
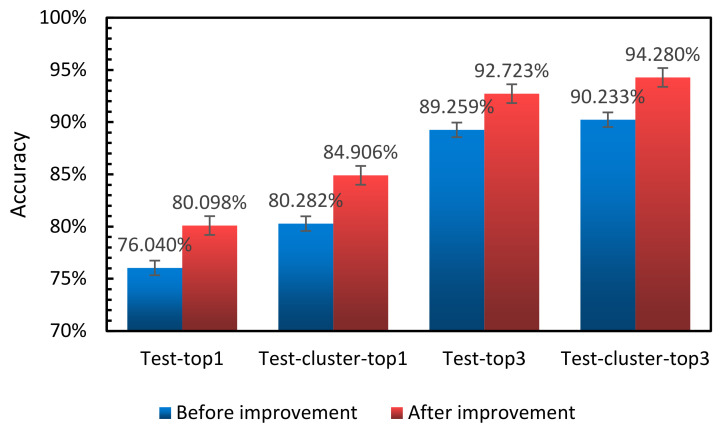
Comparison of accuracy classification prediction of the model before and after optimization.

As shown in Figure 12, top1 increased by 4.58%, and top3 increased by 4.624%. After K-means clustering, the accuracy of top1 increased by 3.64% on average, and the accuracy of top3 classification increased by 4.047%. Experimental results were better than those before, which shows that our optimization of the model is effective.

### 3.5. Result Comparison and Analysis

We compared proposed model ResNet10-v1 with other advanced tactile recognition models, such as ResNet18 [14] and ResNet50. Classification accuracy is listed in Table 2 and Table 3, and our model obviously achieved the best performance.

**Figure 13:**
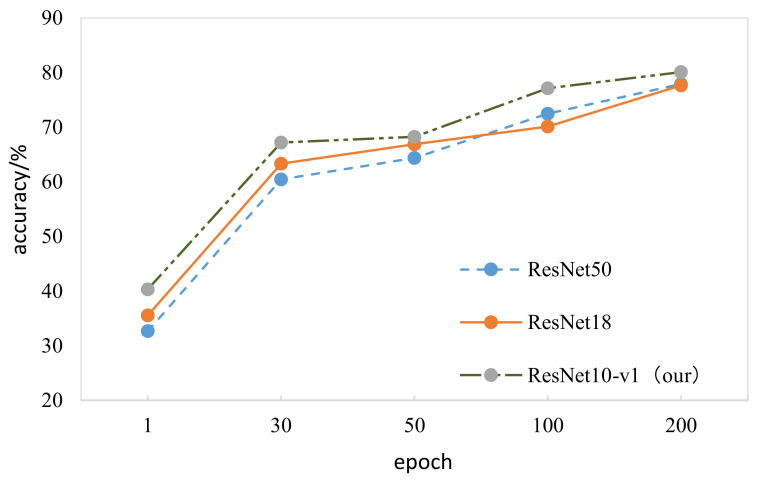
Comparison of ResNet10-v1, ResNet18, and ResNet50 model classification prediction accuracy.

Figure 13 shows the average accuracy of target classification obtained in different epochs; the accuracy of our optimized model was higher than that of the two other residual network models.

In addition, we compared work related to the research content of this paper in recent years, and results are shown in Table 4.

Table 4 shows that the test time of our model was better than that of some models proposed in recent years. Our model is more lightweight than current advanced convolutional neural networks ResNet18, ResNet50, and Vgg16, which lays the foundation for subsequent applications and implementations in embedded devices.

## 4. Conclusions

In this paper, we proposed an effective target classification model (ResNet10-v1) based on pure tactile perception data. This model uses the advantages of convolutional neural networks and deep residual networks, reduces the lack of edge features, and improves feature extraction ability in the object classification problem of tactile perception data. By optimizing the proposed model hyperparameters and the number of model input frames, we increased the accuracy of the target with the best classification effect (test-top1) to 80.098%, and the accuracy of the three classes with better classification results (test-top3) to 92.72%. In addition, we processed 32 × 32 tactile-map data through the K-means clustering method and input them into ResNet10-v1, and the object classification effect was further improved. A large number of computational experiments show that our ResNet10-v1 model achieved the best results on pure tactile datasets with complex features compared with popular target classification models ResNet18 and ResNet50. Our method provides an important reference for research in the field of tactile perception. In future work, we aim to further improve the accuracy of pure tactile data classification, and further apply the proposed method to more tactile data with complex characteristics, improving the generalization ability of the method.

## Figures and Tables

**Table 1:** Comparison of ResNet10-v1 model classification prediction accuracy before and after optimization.

	Type	Test-Top1	Test-Cluster-Top1	Test-Top3	Test-Cluster-Top3
Acc (%)	Before	76.040	80.282	89.259	90.233
	After	80.098	84.906	92.723	94.280

**Table 2:** Comparison of ResNet10-v1, ResNet18, and ResNet50 model classification prediction accuracy.

	ResNet50	ResNet18 [14]	ResNet10-v1 (Our)
Test-top1	78.926%	77.671%	80.098%
Test-top3	86.676%	86.793%	92.723%
Test-cluster-top1	81.454%	81.806%	84.906%
Test-cluster-top3	92.112%	91.099%	94.280%

**Table 3:** Comparison of ResNet10-v1, ResNet18, and ResNet50 model classification prediction accuracy.

	ResNet50	ResNet18 [14]	ResNet10-v1 (Our)
1	32.667%	33.554%	40.333%
30	60.445%	63.309%	67.220%
50	64.378%	66.872%	68.233%
100	72.487%	70.129%	77.114%
200	78.926%	77.671%	80.098%

**Table 4:** Comparison results of different classification methods.

Author	Year	Objects	Method	Accuracy	t_GPU_ (s)
Subramanian Sundaram [14]	2014	26	ResNet18	77.67%	3.56
Shan Luo [31]	2015	18	Tactile-SIFT	85.46%	-
Juan M. Gandarias [32]	2019	22	TactNet	93.61%	0.77
Tingting Mi [33]	2021	3	GCN-FF	89.13%	-
Emmanuel Ayodele [34]	2021	6	CNN	75.73%	6.20
Ours	2021	26	ResNet10-v1	80.098%	0.058

## Data Availability

Not applicable.
